# A Ln‐MOF Sensor Based on Dual‐Signal Response and Logic Gating for the Ratio Fluorescence Detection of Anthrax Biomarkers

**DOI:** 10.1002/bio.70493

**Published:** 2026-05-01

**Authors:** Yuhang Zhang, Xiang Hou, Wenwen Deng, Zhixin Wang, QuTong Zheng

**Affiliations:** ^1^ Research Center for Precision Medication of Chinese Medicine, College of Pharmacy Hunan University of Chinese Medicine Changsha China; ^2^ Institute of Traditional Chinese Medicine Health Industry China Academy of Chinese Medical Sciences Nanchang China

**Keywords:** anthrax biomarkers, Ln‐MOF sensor, logic gating, ratiometric fluorescence sensor

## Abstract

Anthrax is an acute infectious disease caused by *Bacillus anthracis* . It can damage the skin, lungs, and intestines of humans and animals and can be fatal in severe cases. Therefore, it is of great significance to develop highly efficient detection methods. Pyridine‐2,6‐dicarboxylic acid (DPA), a major component of bacterial spores, can be used as a biomarker for the detection of anthrax. However, existing biological and chemical detection methods have shortcomings. Biological methods often involve lengthy procedures, complex equipment, and expensive reagents. In chemical methods, most fluorescence sensors for DPA detection have low sensitivity, precision, and accuracy. To solve this, a dual‐signal‐responsive ratiometric fluorescence sensor was designed. First, Bio‐MOF was synthesized with Zn^2+^, adenine, and 1,3,5‐benzenetricarboxylic acid. Then, Tb^3+^ was introduced via cation exchange, and 1‐Hydroxypyrene (1‐OHP) was introduced via hydrogen bonds to get 1‐OHP/Tb@Bio‐MOF. With DPA, Tb^3+^ forms a complex, enhancing its emission (“turn‐on”), while DPA reduces 1‐OHP emission (“turn‐off”) via IFE and PET. This sensor detects DPA in serum sensitively and selectively, and a DPA‐monitoring logic gate was made. Also, a paper‐based sensor (1‐OHP/Tb‐MOF@CP) was prepared by blending.

## Graphical abstract

The dual‐emission MOF composite (1‐OHP/Tb@Bio‐MOF)‐based fluorescence sensing system for DPA detection involves (a) preparation of the dual‐emission MOF composite; (b) investigation of fluorescence sensing performance; (c) elucidation of the fluorescence response mechanism of 1‐OHP/Tb@Bio‐MOF to DPA; and (d) detection and application of DPA based on the dual‐signal sensor.

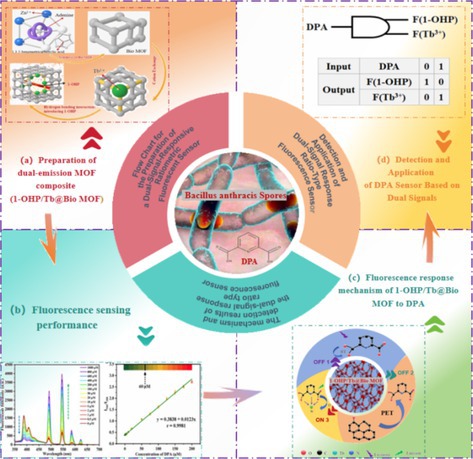

## 1 Introduction

Anthrax is an acute zoonotic infectious disease caused by *Bacillus anthracis* . It is classified as a Class B infectious disease in China and can damage the skin, lungs, and intestines of humans and animals. For cutaneous anthrax, the symptoms include black eschar, tissue edema, pain, and fever. In severe cases, it can lead to sepsis and meningitis. Pulmonary anthrax initially resembles a cold but soon progresses to severe dyspnea, cyanosis, and hemoptysis, often accompanied by sepsis and shock, and can be fatal in a short time. Intestinal anthrax presents as gastrointestinal inflammation, with symptoms such as nausea, vomiting, abdominal pain, diarrhea, and bloody stools. In severe cases, it can cause intestinal perforation and peritonitis and subsequently lead to sepsis and shock [1, 2]. When preventing and controlling anthrax, it is of crucial importance to develop highly efficient and ultrasensitive detection methods. Pyridine‐2,6‐dicarboxylic acid (DPA), a key component of bacterial spores, accounts for approximately 5%–15% of the dry weight of spores and can be released through physical, chemical lysis, or germination [3]. Based on this characteristic, DPA can serve as a biomarker for the detection of anthrax, facilitating the detection of this disease.

In recent years, a variety of biological and chemical methods have been used to monitor DPA. Biological methods such as PCR [4] and immunoassays [5] have lengthy procedures, complex equipment, and expensive reagents, making them unsuitable for on‐site monitoring [6]. The chemical methods mainly include surface‐enhanced Raman spectroscopy [7] and photoluminescence spectroscopy [8], whereas fluorescence detection is favored due to its low cost and fast response [9]. However, most fluorescence sensors have single‐signal responses, resulting in poor sensitivity and other issues [10]. Developing self‐calibrating fluorescent logic devices is of great significance for accurate monitoring [6, 11]. In addition, this type of fluorescent sensor can achieve Boolean logic gate operations through the combination of optical and chemical inputs and outputs. It can transmit binary and multi‐valued information, much like the human brain [12]. In many fields such as biomedical diagnosis and drug delivery [6, 13, 14], luminescence‐based logic‐gate intelligent materials have been designed and applied.

To overcome the challenges in DPA detection, researchers have designed and synthesized a variety of luminescent materials [15, 16, 17]. Among them, Ln‐MOFs have attracted significant attention in the field of chemical sensing due to their combination of the structural advantages of MOFs and the unique photoluminescence properties of lanthanide elements [18, 19]. However, most of the existing Ln‐MOFs‐based DPA sensors are single emission and are easily affected by external factors [3]. Although ratio fluorescence sensors based on two independent emissions can effectively circumvent this problem, judging from the currently published research results, such sensors generally use one fluorescence emission as an internal standard, and the other fluorescence emission responds in an “on” or “off” form [20, 21, 22]. The design of such sensors with dual‐signal responses still needs to be broken through, and making them into thin‐film types can give full play to their greater advantages [6, 23].

This paper aims to rapidly, sensitively, and accurately detect DPA. A dual‐signal‐responsive ratiometric fluorescence sensor based on the “turn‐on” fluorescence of Tb@Bio‐MOF and the “turn‐off” fluorescence of 1‐OHP is designed. A dual‐emission MOF composite material is synthesized through specific steps. Based on the dual‐fluorescence‐signal‐response mechanism, a sensor is constructed, a logic gate is designed, and a paper‐based sensor is prepared.

## 2 Experimental Section

### 2.1 Materials

Adenine (Ade) and 1,3,5‐benzenetricarboxylic acid (H_3_BTC) were purchased from Shanghai Macklin Biochemical Technology Co. Ltd. Terbium nitrate hexahydrate (Tb(NO_3_)_3_·6H_2_O) and DPA were obtained from Saan Chemical Technology (Shanghai) Co. Ltd. 1‐Hydroxypyrene (1‐OHP), anhydrous zinc chloride (ZnCl_2_), sodium formate, N,N‐dimethylformamide (DMF), ethanol, and so forth were all purchased from Sinopharm Chemical Reagent Co. Ltd. All of the above reagents were of analytical grade and did not require further purification. Cellulose pulp was purchased from Taobao. Cellulose filter paper (Shuangquan qualitative filter paper) was bought from GE Healthcare Bio‐Sciences (Hangzhou) Co. Ltd. Deionized water was prepared using a Millipore instrument system (Molsheim, France). A 10‐mM DPA stock solution was prepared by dissolving 167 mg of DPA in 100 mL of a NaOH solution (20 mM), and DPA solutions of other concentrations were prepared by diluting the stock solution with deionized water.

### 2.2 Instrument

The particle size distribution of the materials was obtained using a Zetasizer Nano ZSP nanoparticle size analyzer (Malvern Instruments Ltd., UK). The powder X‐ray diffraction (PXRD) patterns were obtained on a Bruker D8 diffractometer (Bruker AXS, Germany), using a copper target (40 mA, 40 kV) for the measurement. The XPS spectra were obtained by a Thermo Scientific K‐Alpha X‐ray photoelectron spectrometer (Thermo Fisher Scientific, USA). The excitation and emission spectra were measured by a Hitachi F‐4600 fluorescence spectrophotometer (Hitachi High‐Technologies Corporation, Japan), using a 450‐W xenon lamp as the excitation source. The Fourier transform infrared (FT‐IR) spectra were obtained on a Thermo Nicolet NEXUS 470 infrared spectrometer (Thermo Fisher Scientific, USA), determined by the method of grinding and pressing the material with potassium bromide. The ultraviolet–visible absorption spectra were measured using a Shimadzu UV‐2600 ultraviolet spectrophotometer (Shimadzu Corporation, Japan). The scanning electron microscope (SEM) images were obtained on a JSM‐6510 SEM (JEOL Ltd., Japan). The ultrasonic heating was carried out using a JYD‐650 intelligent ultrasonic cell crusher (Shanghai Zhixin Instrument Co. Ltd., China). The microwave heating was conducted using an XH‐100B microwave catalytic synthesis/extraction instrument (Hebei Xianghu Scientific Instrument Co. Ltd., China).

### 2.3 Preparation of Bio‐Metal–Organic Framework (Bio‐MOF)

Please refer to Supporting Information S1 for details.

### 2.4 Preparation of Tb@Bio‐MOF

Tb@Bio‐MOF was prepared through a cation‐exchange reaction, in which the dimethylamine cations (DMA^+^) in Bio‐MOF were replaced by Tb^3+^ [24]. The specific procedures are as follows: Immerse Bio‐MOF in a DMF solution containing 0.1 M Tb(NO_3_)_3_·6H_2_O for 4 h. After the immersion, wash the resulting product three times with ethanol, and then dry it in a vacuum drying oven at 55°C for 12 h.

### 2.5 Preparation of 1‐OHP/Tb@Bio‐MOF Composite

The 1‐OHP/Tb@Bio‐MOF composite was prepared by post‐synthetic modification of MOF. The specific steps are as follows: Accurately weigh 200 mg of Tb@Bio‐MOF and 0.1 mg of 1‐OHP, and disperse them in 20 mL of methanol. Stir the mixture at room temperature for 3 h. Subsequently, place it in an environment of 40°C and remove the solvent by vacuum rotary evaporation. Finally, dry the obtained white powder overnight in a vacuum at 45°C.

### 2.6 Preparation of the Paper‐Based Sensor (1‐OHP/Tb‐MOF@CP)

First, prepare the pulp suspension: Under magnetic stirring, disperse the cellulose pulp in 10 mL of deionized water at a concentration of 0.5% (g/mL) to obtain the pulp suspension. Next, disperse the composite material: Accurately weigh 2.5 mg of 1‐OHP/Tb@Bio‐MOF and add it to the prepared pulp suspension. Then, perform ultrasonic treatment for 5 min to ensure that the 1‐OHP/Tb@Bio‐MOF composite material is uniformly dispersed in the pulp suspension. Then, carry out the vacuum filtration step: Prepare a sand‐core funnel of model G3 with a diameter of 4 cm, and place a cellulose filter paper of the same size on the sand core. Pour the pulp suspension containing 1‐OHP/Tb@Bio‐MOF into the sand‐core funnel and perform vacuum filtration to finally obtain a circular MOF‐composite cellulose paper‐based membrane. Finally, process the paper‐based membrane: Carefully transfer the obtained paper‐based membrane between two glass plates and gently press it by hand for 5 min to make the paper membrane more compact, uniform, and flat. After pressing, place the paper‐based membrane in a vacuum environment at 65°C and dry it for 24 h. The 1‐OHP/Tb‐MOF@CP composite cellulose paper‐based membrane is prepared.

### 2.7 Experimental Operations for Fluorescent Sensing of DPA

Please refer to Supporting Information S2 for details.

### 2.8 Detecting DPA in Serum

Please refer to Supporting Information S3 for details.

### 2.9 Computational Methods

Please refer to Supporting Information S4 for details.

## 3 Results and Discussion

### 3.1 Preparation of 1‐OHP/Tb@Bio‐MOF

Bio‐MOFs are constructed using biomolecules as bridging ligands and possess excellent properties such as environmental friendliness, good biocompatibility, and tunable pore sizes. Therefore, they were selected as the carriers for lanthanide metal ions in this experiment. The dispersion degree of MOF particles in liquids is closely related to their particle size: The smaller the particle size, the easier it is to achieve uniform dispersion. Thus, the synthesis conditions of Bio‐MOFs, including solvent dosage, heating temperature, and heating method, were optimized at the initial stage of the experiment.

According to the experimental results in Table S1, when the volume ratio of DMF/H_2_O is 36 mL/9 mL, the particle size of the prepared Bio‐MOF reaches the minimum value, with an average particle size of 474 nm, as shown in Figure S1a. This phenomenon can be attributed to the influence of the change in reactant concentration on the nucleation process of MOF. As the reactant concentration decreases, the number of nuclei formed per unit volume increases, resulting in a decrease in the particle size of the generated MOF [25].

However, when the reactant concentration is further decreased (such as in Conditions 3 and 4), the particle size of Bio‐MOF increases instead. By analyzing its PXRD pattern, it is found that it does not match the simulated PXRD pattern, indicating that the structure of Bio‐MOF may have changed, thus leading to the change in particle size.

It can be seen from Conditions 1, 5, and 6 in Table S1 that the heating temperature also has a significant impact on the particle size of Bio‐MOF. As the heating temperature decreases, the particle size of Bio‐MOF gradually increases. This is because a decrease in temperature slows down the nucleation and growth rates of MOF [26].

In addition, by comparing Conditions 1, 7, and 8 in Table S1, the conventional heating method is superior to microwave heating and ultrasonic heating in terms of particle size control. Because it is difficult to precisely control the heating time in microwave and ultrasonic heating, the particle size is relatively large [27].

Taking into account the influence of various factors on the particle size of Bio‐MOF, Condition 2 in Table S1 is selected as the optimal synthesis condition for Bio‐MOF to ensure the preparation of Bio‐MOF materials with a smaller particle size and stable structure.

Subsequently, the immersion time of Bio‐MOF in the Tb^3+^ DMF solution and the mass ratio of 1‐OHP to Tb@Bio‐MOF were optimized. As shown in Figure S1b, the fluorescence intensity reached its maximum when the immersion time was 4 h and then tended to be constant, indicating that the cation exchange between Bio‐MOF and Tb^3+^ reached saturation. Figure S2a–e shows the emission spectra of the 1‐OHP/Tb@Bio‐MOF composites synthesized under five different mass ratios in water and an aqueous DPA solution (1 mM). Evidently, the addition of DPA led to a decrease in the fluorescence of 1‐OHP at 388 nm and an increase in the fluorescence of Tb@Bio‐MOF at 546 nm. It can be seen from Figure S2f that when the mass ratio was 1/2000, the chromaticity shift value was the largest, suggesting that the composite was the most sensitive to DPA. The chromaticity shift value here refers to the distance between the color coordinates of 1‐OHP/Tb@Bio‐MOF in water and the aqueous DPA solution. The larger the distance, the more sensitive the material is to DPA. Therefore, an immersion time of 4 h and a mass ratio of 1‐OHP to Tb@Bio‐MOF of 1/2000 were selected as the optimal conditions.

### 3.2 Characterization of 1‐OHP/Tb@Bio‐MOF

**FIGURE 1:**
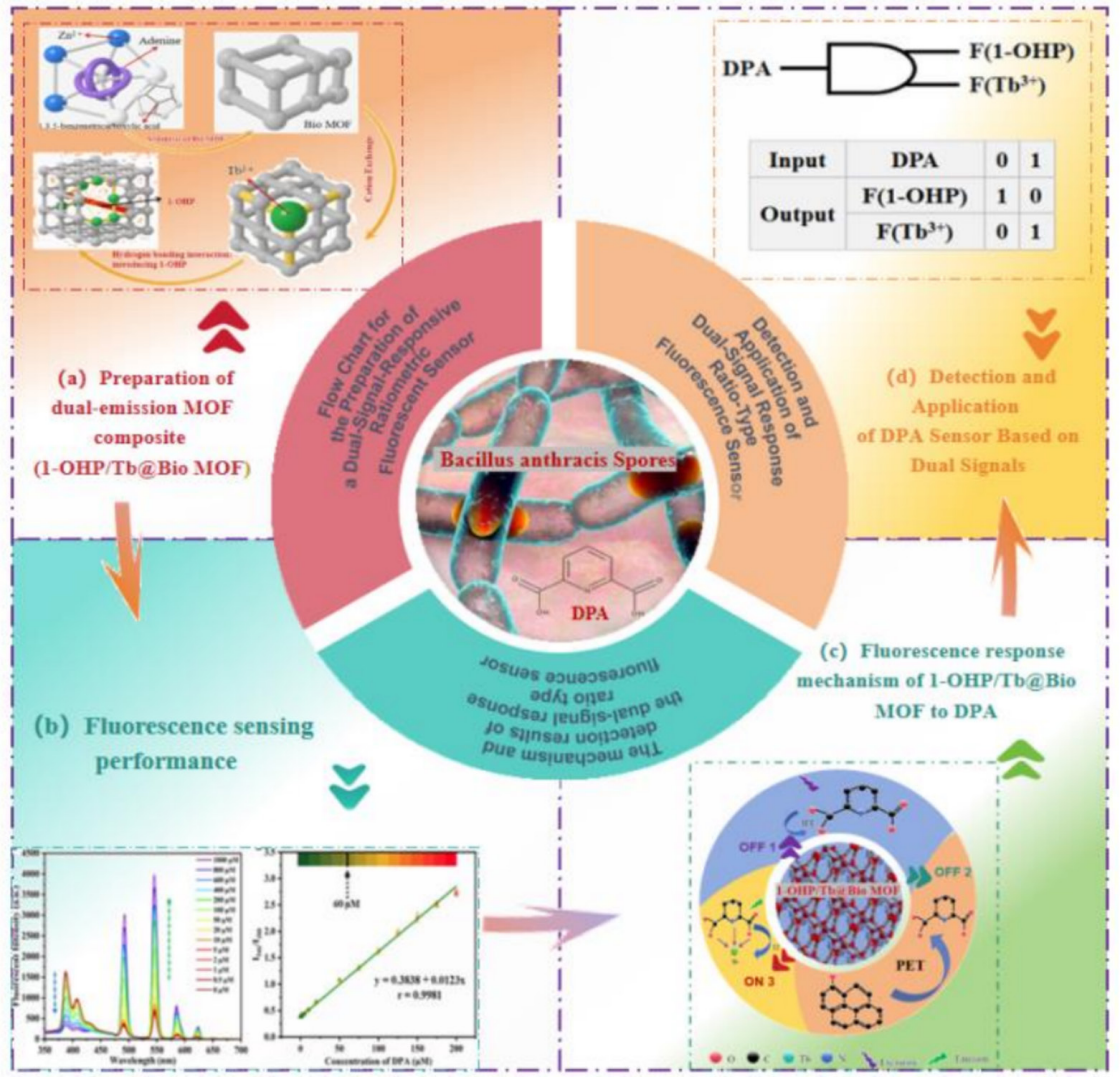
Schematic illustration of the dual‐emission MOF composite (1‐OHP/Tb@Bio‐MOF)‐based fluorescence sensing system for DPA detection. It encompasses four key parts: (a) preparation of the dual‐emission MOF composite; (b) investigation of fluorescence sensing performance; (c) elucidation of the fluorescence response mechanism of 1‐OHP/Tb@Bio‐MOF to DPA; and (d) detection and application of DPA based on the dual‐signal sensor.

**FIGURE 2:**
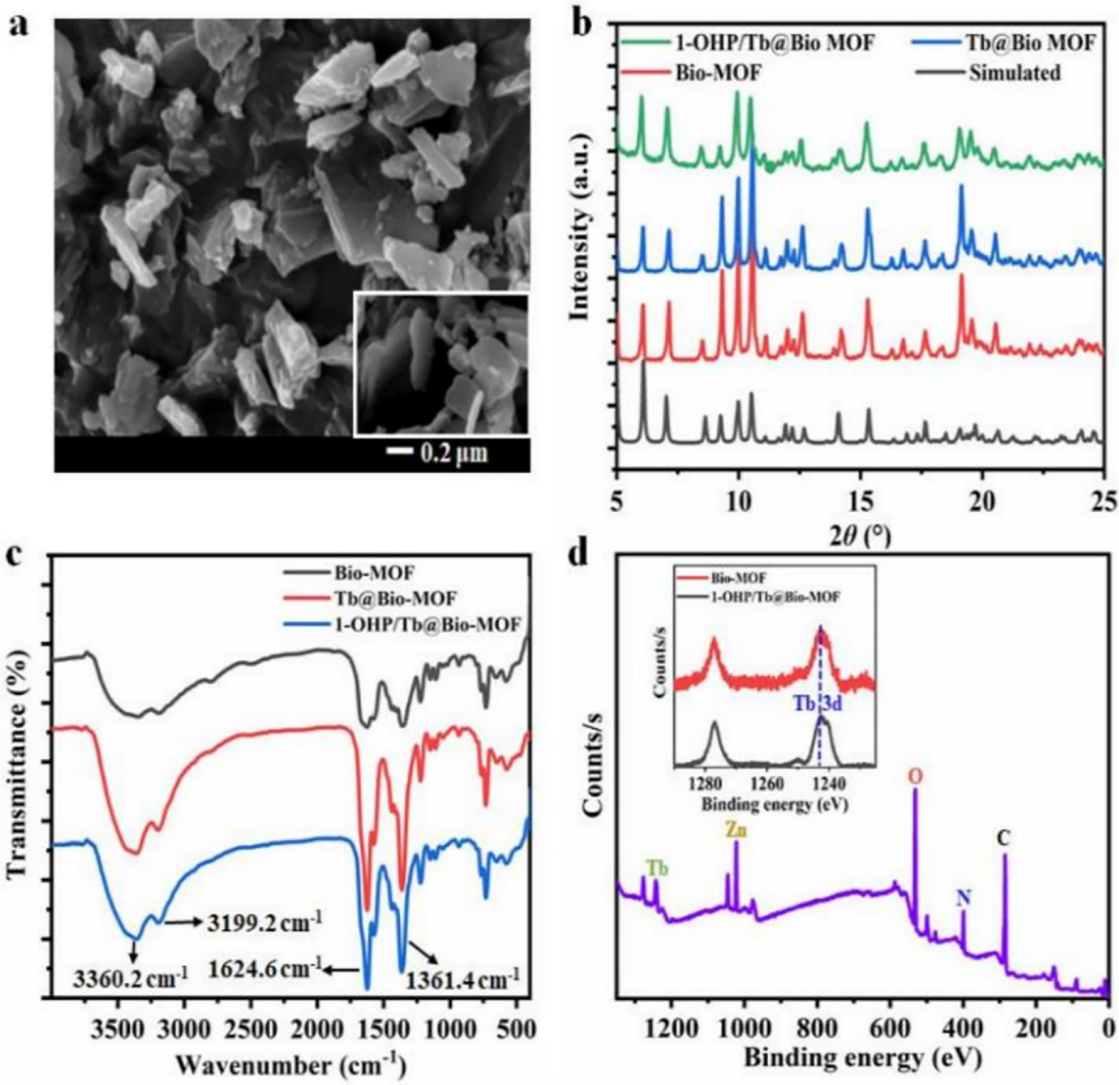
(a) SEM image of 1‐OHP/Tb@Bio‐MOF (Inset: SEM image of Bio‐MOF); PXRD patterns (b) and infrared spectra (c) of Bio‐MOF, Tb@Bio‐MOF, and 1‐OHP/Tb@Bio‐MOF; and (d) XPS spectrum of 1‐OHP/Tb@Bio‐MOF, Inset: Tb 3d XPS spectra of Tb@Bio‐MOF and 1‐OHP/Tb@Bio‐MOF.

The SEM results in Figure 1a show that both Bio‐MOF and 1‐OHP/Tb@Bio‐MOF are irregular flake‐shaped crystals (particle size: 200–1000 nm). The former has a smooth surface, whereas the latter becomes rough after modification with Tb^3+^ and 1‐OHP, with its lattice unaffected. The PXRD pattern comparison in Figure 2b further verifies that the patterns of Bio‐MOF, Tb@Bio‐MOF, and 1‐OHP/Tb@Bio‐MOF are highly consistent, almost overlapping with the simulated pattern (CCDC: 1507714), and no new diffraction peaks appear [28].

In conclusion, SEM and PXRD jointly confirm that the modification with Tb^3+^ and 1‐OHP does not change the crystal structure of Bio‐MOF, providing a basis for the structural stability of subsequent research and applications.

Figure 2c (FT‐IR spectra) shows that Bio‐MOF, Tb@Bio‐MOF, and 1‐OHP/Tb@Bio‐MOF have similar absorption bands (1624.6 and 1361.4 cm^−1^ for —COO^−^ stretching and 3360.2 and 3199.2 cm^−1^ for O—H and N—H stretching, respectively), indicating that Tb^3+^/1‐OHP modification does not alter Bio‐MOF's chemical bonding. Figure 1d (XPS) reveals that 1‐OHP/Tb@Bio‐MOF has a Tb 3d peak (confirming Tb^3+^ incorporation) and that the Tb 3d peaks (1242.7 eV) of Tb@Bio‐MOF and 1‐OHP/Tb@Bio‐MOF coincide (similar Tb^3+^ binding energy/electron density) [3]. Per Reference [28], this Bio‐MOF is stable at 420°C (good high‐temperature resistance) and has a suitable pore size (15.2 Å × 10.5 Å) to allow DPA entry while restricting macromolecules like proteins.

### 3.3 Luminescence Properties of 1‐OHP/Tb@Bio‐MOF

Figure S3 presents the fluorescence excitation and emission spectra of 1‐OHP, Tb@Bio‐MOF, and 1‐OHP/Tb@Bio‐MOF. The excitation spectrum of 1‐OHP/Tb@Bio‐MOF has two distinct absorption bands (210–310 nm) with peaks at 240 and 297 nm, which are ascribed to the π–π* electronic transitions of 1‐OHP in the composite and BTC ligands in Tb@Bio‐MOF [3]. Under 278‐nm excitation, 1‐OHP shows fluorescence emission at 370–450 nm (maximum at 388 nm), whereas Tb@Bio‐MOF and 1‐OHP/Tb@Bio‐MOF exhibit different emission characteristics. Tb@Bio‐MOF has sharp emission bands at 490, 544, 585, and 620 nm, corresponding to the ^5^D_4_‐^7^F_j_ (*J* = 6, 5, 4, and 3) transitions of Tb^3+^, respectively [29]. The 1‐OHP/Tb@Bio‐MOF, more notably, has five emission peaks at 388, 497, 546, 591, and 627 nm. It is worth noting that the emission peak of 1‐OHP at 388 nm is not interfered with by the characteristic emissions of Tb^3+^. Based on the above results, it can be clearly concluded that this nanocomposite has the fluorescence property of single excitation and dual emission, which provides unique advantages for its applications in related fields.

#### 3.3.1 Stability of the DPA Sensor

**FIGURE 3:**
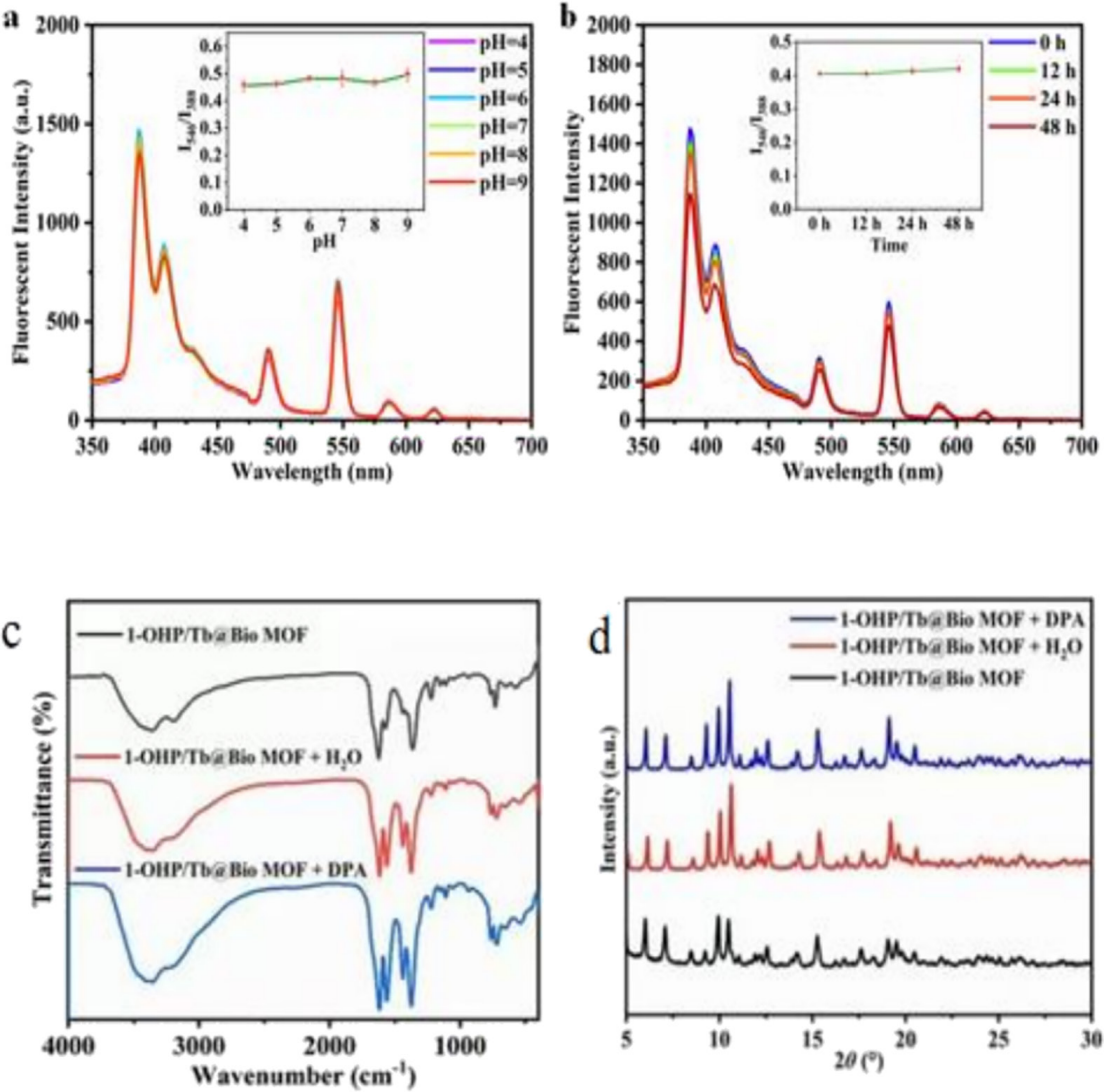
Fluorescence spectra of 1‐OHP/Tb@Bio‐MOF in aqueous solutions with different pH values (a) and at different times (b) (λ_ex_ = 278 nm, scanning range: 350–700 nm). The insets show the changes in the fluorescence intensity ratio (I_546_/I_388_) with pH values and time. (c) Infrared spectra and (d) PXRD patterns of 1‐OHP/Tb@Bio‐MOF, 1‐OHP/Tb@Bio‐MOF in contact with water, and 1‐OHP/Tb@Bio‐MOF in contact with DPA.

Fluorescence stability is critical for fluorescence sensors [3], yet most reported sensors only maintain stable luminescence in a narrow pH range [30]. This experiment tested 1‐OHP/Tb@Bio‐MOF's fluorescence stability by measuring its dispersion's emission spectra at different pH (4–9, matching biological samples' pH 4.5–8.0 [31]) and time points: Figure 3a shows unchanged fluorescence spectra and I_546_/I_388_ ratio (pH 4–9, good pH stability); Figure 3b shows no obvious changes in these parameters after 48‐h storage (good temporal stability). Additionally, FT‐IR and PXRD (Figure 3) confirmed that its crystal structure remains intact in water and aqueous DPA solution, indicating that 1‐OHP/Tb@Bio‐MOF is water compatible with good photostability and structural stability in aqueous environments—beneficial for DPA sensing in aqueous systems.

### 3.4 Ratiometric Fluorescence Sensing of DPA by 1‐OHP/Tb@Bio‐MOF

#### 3.4.1 Fluorescence Sensing Performance

**FIGURE 4:**
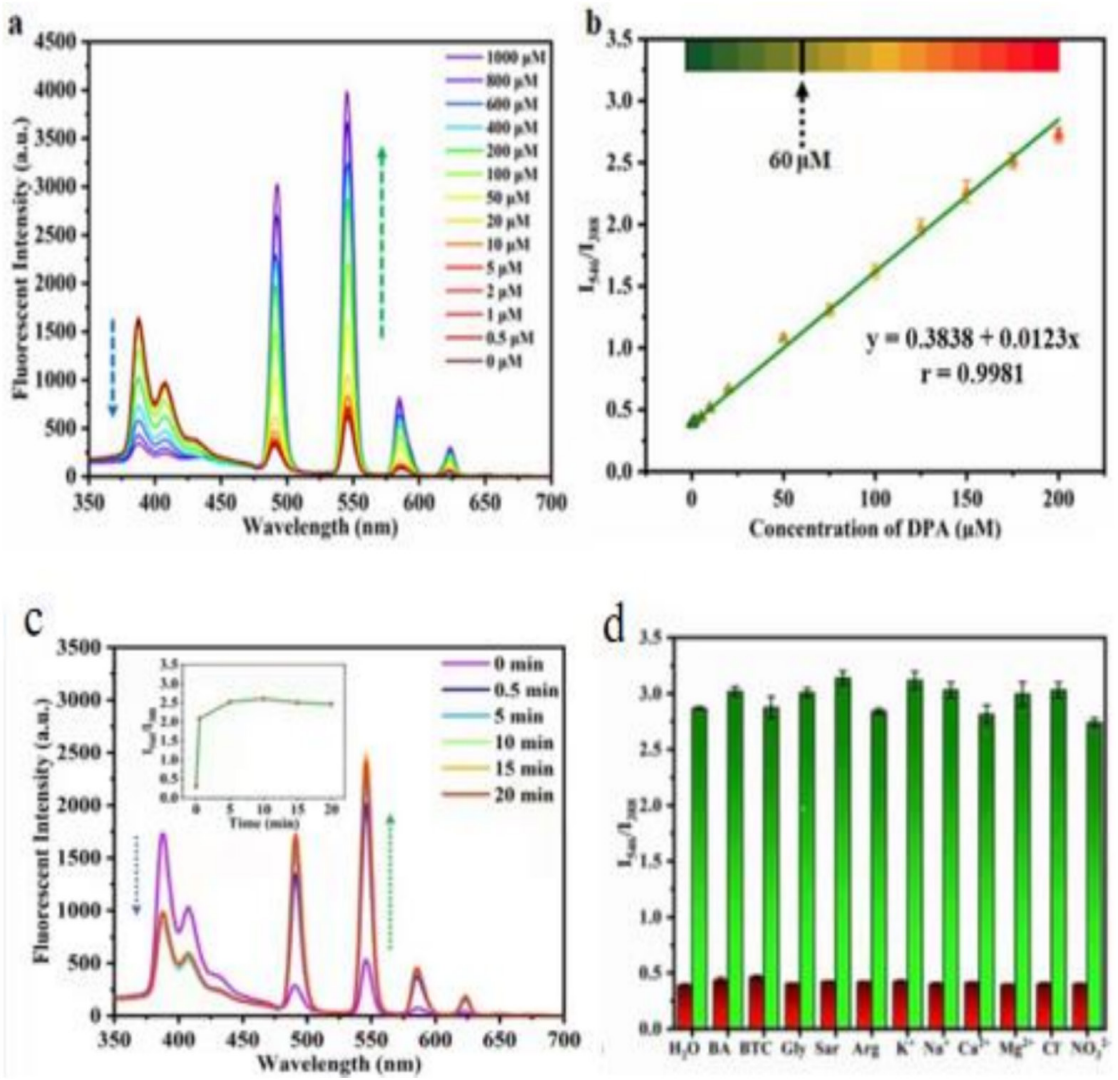
(a) Fluorescence spectra of 1‐OHP/Tb@Bio‐MOF in aqueous DPA solutions with different concentrations. (b) The relationship curve between the fluorescence intensity ratio (I_546_/I_388_) of 1‐OHP/Tb@Bio‐MOF and the DPA concentration (λ_ex_ = 278 nm, scanning range: 350–700 nm). (c) Fluorescence emission spectra at different times after adding DPA to the aqueous dispersion of 1‐OHP/Tb@Bio‐MOF. (d) Selectivity of 1‐OHP/Tb@Bio‐MOF in response to DPA (λ_ex_ = 278 nm, scanning range: 350–700 nm). The concentrations of both DPA and interfering substances are 200 μM; green fluorescent part: H_2_O/interfering substances, red fluorescent part: DPA/(interfering substances + DPA). Arg: arginine, BA: benzoic acid, BTC: trimesic acid, Gly: glycine, Sar: sarcosine.

Figure 4a shows 1‐OHP/Tb@Bio‐MOF's emission spectra in aqueous DPA solutions of different concentrations: As DPA concentration rises, its fluorescence intensity at 388 nm decreases, whereas that at 546 nm increases—this dual‐signal response gives it a self‐calibration function for ratiometric fluorescence quantitative analysis, which avoids fluctuations/errors (common in single‐emission methods [6, 32]) to improve measurement precision, accuracy, and sensitivity. Figure 4b displays the linear relationship between fluorescence intensity ratio (I_546_/I_388_) and DPA concentration (0–200 μM), with the linear regression equation *y* = 0.3838*x* + 0.0123 (*r* = 0.9981, *y* = I_546_/I_388_, *x* = DPA concentration); the LOD (calculated via 3S/N, S = I_546_/I_388_ at 2‐μM DPA, *N* = 6‐time baseline noise standard deviation) is 0.4 μM. This LOD is lower than most other fluorescent probes and far below the minimum anthrax spore infectious dose for humans (60 μM [33]), showing high application potential in DPA detection.

#### 3.4.2 Response Time and Selectivity

The rapid sensor response to dipicolinic acid (DPA) favors real‐time analysis [34]. The 1‐OHP/Tb@Bio‐MOF sensor's response time was tested via the in situ luminescence method [2]: After adding DPA to its aqueous dispersion, obvious emission spectrum changes (388‐nm peak decrease and 546‐nm peak increase) were observed within 0.5 min, and the response stabilized at 5 min (I_546_/I_388_ became constant). Thus, this fluorescence sensor shows fast DPA response, highly suitable for real‐time monitoring to provide timely and accurate DPA information.

In real detection scenarios, complex sample matrices increase detection difficulty [35], making selectivity critical for fluorescence sensors [36]. To test 1‐OHP/Tb@Bio‐MOF's DPA selectivity (Figure 4d), interfering substances—DPA structural analogs (e.g., benzoic acid and trimesic acid), common biological amino acids (glycine, sarcosine, and arginine), and anions/cations (K^+^, Na^+^, Ca^2+^, Mg^2+^, Cl^−^, and NO_3_^2−^)—were used. Results showed that these interferents barely affected the sensor's I_546_/I_388_ ratio, and coexistence with DPA also did not impair DPA‐specific recognition. Thus, the sensor has high DPA selectivity and anti‐interference ability, likely due to DPA's structure: Nitrogen in its pyridine moiety and oxygen in carboxyl groups form a strong coordination affinity with Tb^3+^ [37, 38], enabling accurate DPA recognition in complex matrices.

### 3.5 Sensing Mechanism of DPA

**FIGURE 5:**
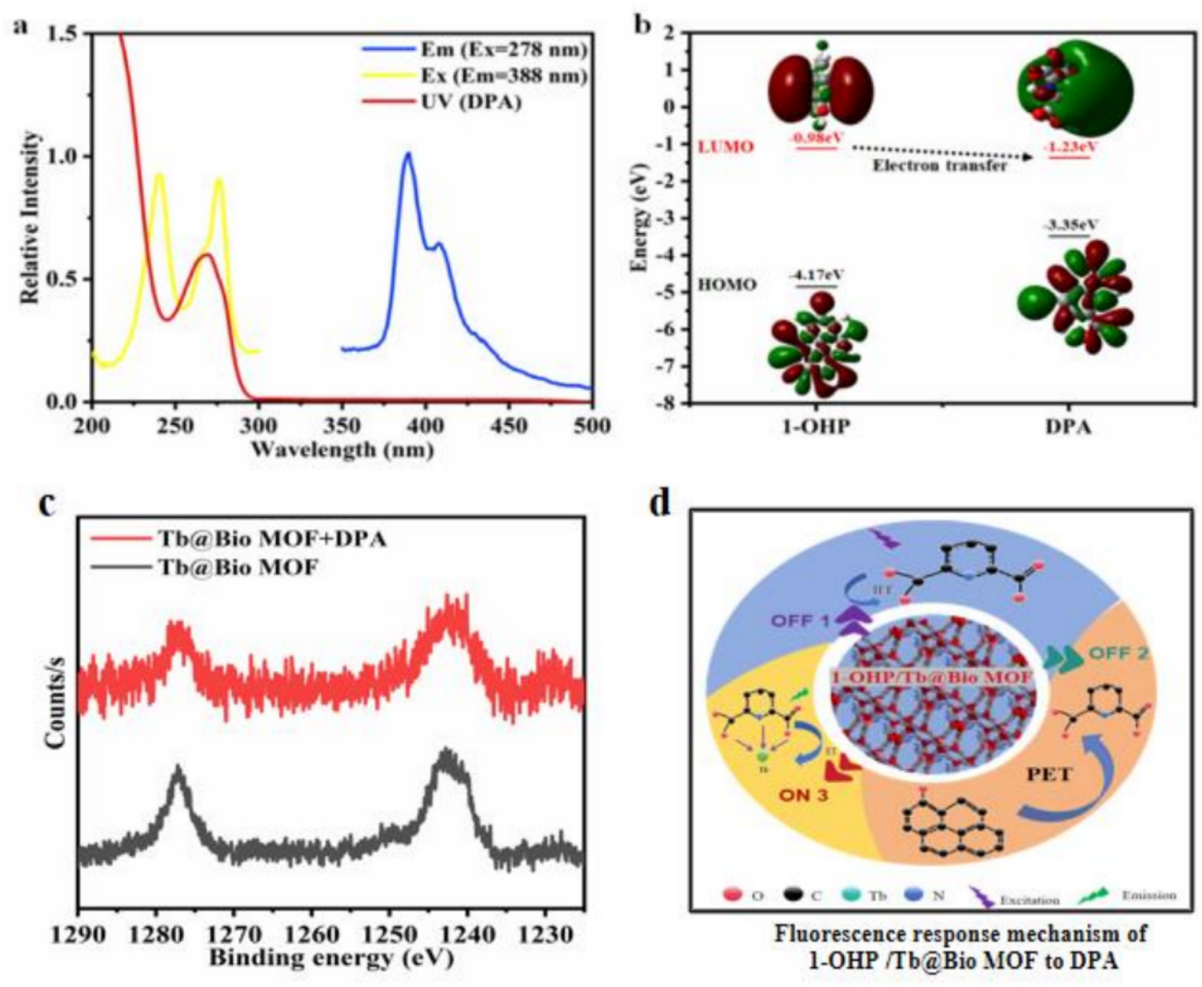
(a) Excitation and emission spectra of a 1‐OHP methanol solution (0.05 mM) (λ_ex_ = 278 nm, scanning range: 200–500 nm) and the UV absorption spectrum of a DPA methanol solution (0.2 mM). (b) HOMO and LUMO energy levels of 1‐OHP and DPA. (c) Tb 3d XPS spectra of Tb@Bio‐MOF and Tb@Bio‐MOF in contact with DPA. (d) Fluorescence response mechanism of 1‐OHP/Tb@Bio‐MOF to DPA.

To clarify DPA‐induced 1‐OHP fluorescence quenching mechanisms, FRET [39], PET [40], and IFE [30] were explored. First, Figure 5a shows no overlap between DPA's UV absorption and 1‐OHP's emission spectra, excluding FRET (per FRET principles). However, DPA and 1‐OHP's absorption spectra mostly overlap around 275 nm, causing competitive excitation light absorption—reducing 1‐OHP's absorbed energy and fluorescence, reflecting IFE. Further, Figure 5b shows that 1‐OHP's LUMO energy (−0.98 eV) is higher than DPA's (−1.23 eV); photo‐excited electrons in 1‐OHP thus transfer from its LUMO to DPA's, confirming PET. In conclusion, DPA‐induced 1‐OHP (388 nm) fluorescence quenching mainly involves IFE and PET, providing a theoretical basis for understanding the sensing system's mechanism.

DPA (with a pyridine ring and two carboxyl groups) complexes rapidly with Tb^3+^ in 1‐OHP/Tb@Bio‐MOF dispersion to form Tb‐DPA complexes [3]; DPA in the complex acts as an “antenna” to absorb excitation energy and transfer it to Tb^3+^, enhancing Tb^3+^ fluorescence [20]. XPS (Figure 5c) confirms this complexation: After DPA addition, Tb^3+^'s 3d peak shifts from 1242.8 to 1240.2 eV (lower energy), due to Tb–O/Tb–N coordination bonds increasing Tb^3+^'s electron cloud density and reducing its binding energy [3]. The ratiometric sensor has three detection channels: IFE/PET triggers 1‐OHP fluorescence “turn‐off,” whereas Tb‐DPA complexation triggers Tb^3+^ fluorescence “turn‐on.” In conclusion, the sensor's DPA sensing mechanism mainly involves 1‐OHP fluorescence decrease (“turn‐off”) via IFE/PET and Tb^3+^ fluorescence increase (“turn‐on”) via Tb‐DPA complexation, providing a theoretical basis for understanding its detection performance.

### 3.6 Determination of DPA in Serum

To verify the 1‐OHP/Tb@Bio‐MOF sensor's practical performance, it was used for DPA detection in human serum samples (results in Table S2). Human serum was spiked with low, medium, and high DPA concentrations; the method showed a 98%–100% recovery rate and relative standard deviation (RSD) < 7%. A recovery rate close to 100% indicates high accuracy (accurate DPA quantification), whereas a low RSD reflects good precision (low result dispersion). In conclusion, this fluorescence sensing method exhibits good accuracy and precision for DPA detection in human serum, with potential for practical application in real‐sample DPA quantification.

### 3.7 Comparison With Literature Methods

To evaluate the 1‐OHP/Tb@Bio‐MOF‐based dual‐emission ratiometric fluorescence sensor's DPA detection performance, it was compared with other Ln‐MOF/Ln‐MOF composite‐based single/dual‐emission sensors (Table S3 [41, 42]). In the detection linear range, it has a wider range than most methods (except [22]), enabling accurate DPA detection across a broader concentration range. In detection limit, it has a lower or comparable limit than most methods (except [43, 44]), ensuring higher sensitivity for trace DPA detection. More importantly, versus single‐emission sensors (easily affected by temperature, humidity, and light source fluctuations, thereby reducing accuracy/precision), this dual‐emission sensor measures two emission intensity ratios—offsetting environmental interferences (similar impacts on both peaks cancel out), significantly improving measurement precision/accuracy. In conclusion, this sensor exhibits excellent DPA detection performance and broad application prospects.

### 3.8 Construction of Logic Gates

**FIGURE 6:**
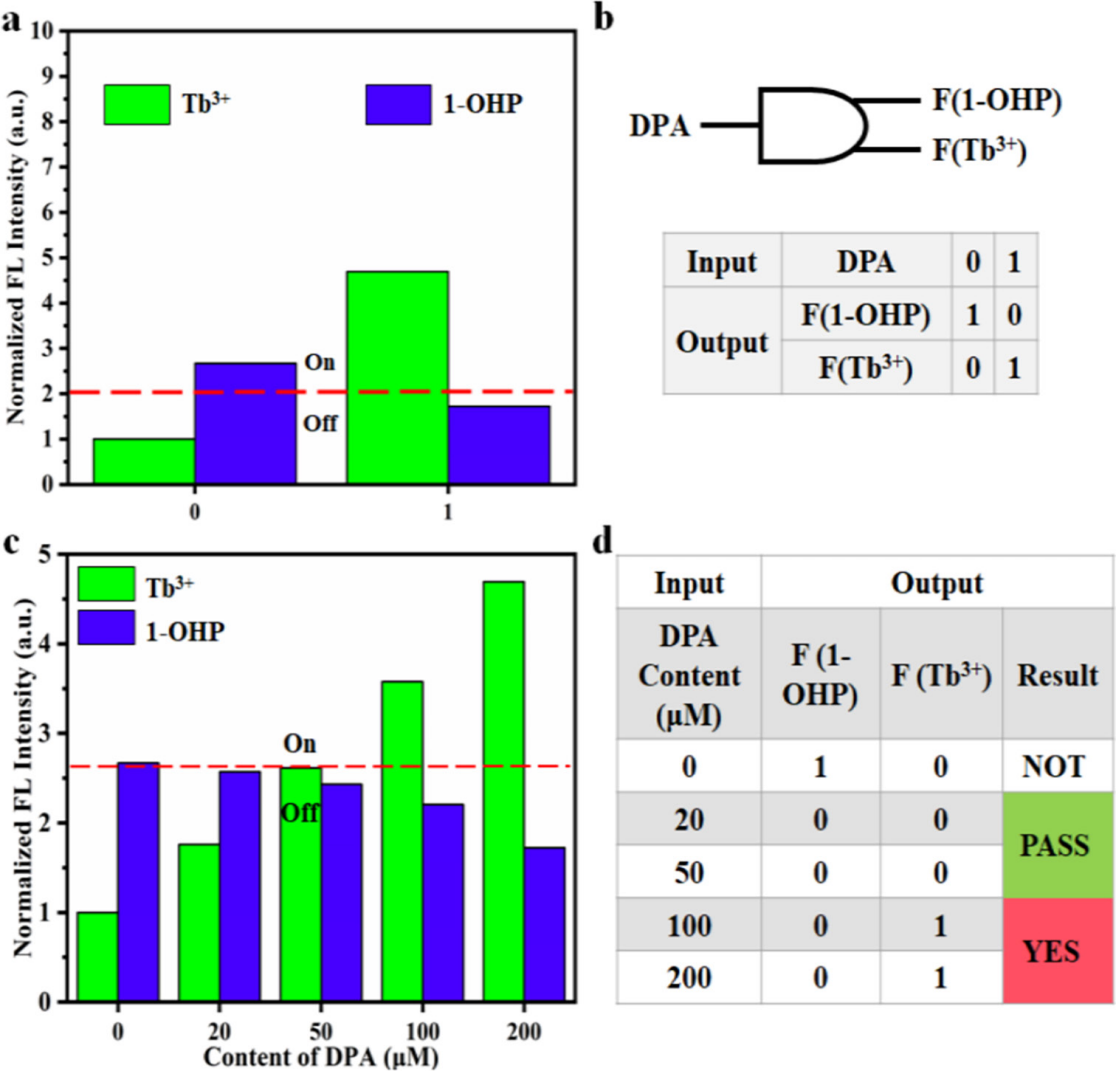
(a, b) Design of the 1–2 decoder logic gate. (c) Normalized fluorescence intensities of 1‐OHP and Tb^3+^ at different DPA concentrations (0–200 μM). (d) Truth table of the logic device for DPA detection.

Given the unique characteristic of the 1‐OHP/Tb@Bio‐MOF sensor, which exhibits dual‐fluorescence signal responses when detecting DPA, it can be used to construct Boolean logic gates for monitoring the concentration level of DPA. Specifically, we constructed a 1–2 logic gate [6] by using the DPA concentration as a single input and the dual‐emission intensities (I_388_ and I_546_) of 1‐OHP/Tb@Bio‐MOF as dual outputs. In the logic setting, the “presence” and “absence” of DPA are defined as “1” and “0” (Input), respectively. The two outputs (Output) correspond to the fluorescence intensities of 1‐OHP (F(1‐OHP)) and Tb^3+^ (F(Tb)), respectively, as shown in Figure 6a,b. When there is no DPA in the system, the dual‐response signals remain stable without any change, and the output of the logic gate at this time is (1, 0). When DPA is present in the system, the fluorescence state of F(Tb) changes from “off” to “on,” whereas the fluorescence state of F(1‐OHP) changes from “on” to “off,” resulting in an output of (0, 1). Figure 6c details the signal output, clearly showing the output responses of the sensor under different input states. We selected five different DPA concentrations within the range of 0–200 μM as input signals and normalized the output signals. As can be seen from Figure 6d, the PASS 0 gate is triggered when the DPA concentration reaches 20 μM, and the YES gate is triggered when the DPA concentration reaches 50 μM. The specific logic output rules are as follows: When the DPA concentration is lower than 20 μM, the logic gate outputs NOT (1, 0); when the DPA concentration is higher than 50 μM, it outputs YES (0, 1); and when the DPA concentration is between 20 and 50 μM, it outputs PASS (0, 0 or 1, 1). In conclusion, we have successfully constructed a 1–2 decoder logic gate. This logic gate can reflect the DPA concentration through four logic groups, namely, NOT (1, 0), PASS 0 (0, 0), PASS 1 (1, 1), and YES (0, 1). Notably, the setting parameters of the logic gate can be manually adjusted. This feature enables the logic gate to flexibly and effectively evaluate the DPA level in the human body according to actual needs.

### 3.9 Construction of Paper‐Based DPA Sensors

Compared with the dispersion sensing mode [3, 6, 22], the paper‐based sensing mode is simpler and faster and can more easily obtain accurate and reliable measurement results. Therefore, in this experiment, a paper‐based sensor (1‐OHP/Tb‐MOF@CP) for DPA detection was prepared. This paper‐based sensor was prepared by a simple blending method. Specifically, 1‐OHP/Tb@Bio‐MOF (0.25 mg/mL) was embedded into cellulose pulp, and the thickness of the paper‐based sensor could be precisely controlled by adjusting the amount of pulp used. During actual use, the paper‐based sensor was cut into a size suitable for a fluorescence cuvette. After directly inserting it into the cuvette and adding the DPA sample solution, measurements could be carried out using a fluorescence spectrophotometer (as shown in Figure S4a). The scanning electron microscopy results (Figure S4b) clearly showed that the 1‐OHP/Tb@Bio‐MOF particles maintained their original morphology and size in the fiber paper and were relatively uniformly distributed on the surface of the cellulose paper. However, when observing the PXRD pattern of 1‐OHP/Tb‐MOF@CP (Figure S4c), only the diffraction peaks of the cellulose paper were present, and the characteristic peaks of 1‐OHP/Tb@Bio‐MOF did not appear. It is speculated that this might be because the content of 1‐OHP/Tb@Bio‐MOF on the surface of the paper‐based sensor was relatively low, and part of the MOF composite material was encapsulated by cellulose, making it difficult for the instrument to detect its diffraction peaks. As shown in Figure S4d, when DPA was present in the system, the intensity of the emission peak of 1‐OHP at 388 nm decreased, whereas the intensity of the characteristic emission peak of Tb^3+^ at 546 nm increased. This phenomenon fully indicated that the combination of 1‐OHP/Tb@Bio‐MOF with cellulose paper did not affect its sensing performance for DPA. In conclusion, the 1‐OHP/Tb‐MOF@CP fiber paper has the potential to be used as a paper‐based fluorescence sensor for DPA detection.

## 4 Conclusions

In this chapter, we successfully prepared the nanocomposite 1‐OHP/Tb@Bio‐MOF using the post‐synthetic modification method and employed it as a ratiometric fluorescence sensor for detecting DPA (a biomarker of anthrax). This sensor features a unique single‐excitation and dual‐emission (turn‐on–turn‐off) luminescent property. Compared with single‐emission sensors, it exhibits higher sensitivity and precision. Based on the dual‐fluorescence signal response mechanism, the sensor achieves highly sensitive and selective detection of DPA. When DPA is present in the system, the 1‐OHP/Tb@Bio‐MOF sensor shows a distinct “turn‐off and turn‐on” dual‐signal fluorescence response. Specifically, the intensity of the emission peak of 1‐OHP at 388 nm decreases, whereas that of Tb^3+^ at 546 nm increases. This phenomenon is attributed to the coordinated operation of three fluorescence detection channels within the sensor. On one hand, the “turn‐off” of 1‐OHP fluorescence is due to the fluorescence inner‐filter effect and photo‐induced electron transfer between DPA and 1‐OHP. On the other hand, the “turn‐on” of Tb^3+^ fluorescence is because DPA molecules form Tb‐DPA complexes with Tb^3+^ in Tb@Bio‐MOF. In this process, DPA molecules act as “antennas” to absorb excitation energy and then sensitize the luminescence of Tb^3+^. To verify the performance of the sensor in real‐sample detection, we applied the 1‐OHP/Tb@Bio‐MOF sensor to the detection of DPA in spiked human serum. The experimental results showed that the recovery rate of the method ranged from 98% to 100%, and the RSD was less than 3%. This fully demonstrates that the sensor has good accuracy and precision and can be used for the quantitative detection of DPA in actual serum samples. In addition, we successfully constructed a 1–2 logic gate and cellulose paper based on the 1‐OHP/Tb@Bio‐MOF sensor for monitoring the DPA level. The logic gate shows different output signals under different DPA concentrations: When there is no DPA, the output signal is NO (1, 0); when the DPA concentration is low, the output signal is PASS (0, 0); and when the DPA concentration is high, the output signal is YES (0, 1). Meanwhile, the 1‐OHP/Tb@Bio‐MOF cellulose paper is simple to prepare and has fluorescence sensing performance for DPA, showing potential as a paper‐based fluorescence sensor for DPA detection. In summary, the 1‐OHP/Tb@Bio‐MOF sensor exhibits excellent performance in DPA detection. It has important application value and development prospects, whether in the quantitative detection of DPA in actual serum samples or in the construction of logic gates and paper‐based sensors for monitoring DPA levels.

## Author Contributions

**Yuhang Zhang:** conceptualization, methodology, data curation, software, investigation, writing – original draft. **Xiang Hou:** methodology, data curation, validation, investigation, software. **Wenwen Deng:** investigation, software. **Zhixin Wang:** investigation, software. **QuTong Zheng:** funding acquisition, supervision, writing – review and editing. The first draft of the manuscript was written by Yuhang Zhang, and all authors commented on previous versions of the manuscript. All authors read and approved the final manuscript.

## Funding

This study was supported by the Natural Science Foundation of Hunan University of Chinese Medicine (2025XJZB002) and the National Natural Science Foundation of China (82304891).

## Conflicts of Interest

The authors declare no conflicts of interest.

## Supporting information


**Table S1:** Optimization of synthesis conditions for Bio‐MOF.
**Figure S1: (a)** Particle size distribution of Bio‐MOF; **(b)** Fluorescence intensity of Bio‐MOF after being immersed in a DMF solution of Tb^3+^(0.1 mM) for different times (500 μg/mL).
**Figure S2:** Emission spectra of 1‐OHP/Tb@Bio‐MOF composites in water and an aqueous DPA solution (1 mM) (λ_ex_ = 278 nm, scanning range: 350–700 nm). Mass ratios of 1‐OHP to Tb@Bio‐MOF used for preparing the composites: **(a)** 1/500; **(b)** 1/1000; **(c)** 1/1333; **(d)** 1/2000; **(e)** 1/5000; **(f)** Chromaticity shift values.
**Figure S3:** Excitation and emission spectra of **(a)** 1‐OHP (0.01 μg/mL), **(b)** Tb@Bio‐MOF (500 μg/mL), and **(c)** 1‐OHP/Tb@Bio‐MOF (500 μg/mL) (λ_ex_ = 278 nm, scanning range: 200–700 nm).
**Table S2:** Determination of DPA in human serum samples (*n* = 3).
**Table S3:** Comparison of various fluorescence sensors for DPA detection.
**Figure S4: (a)** Detection mode of the paper‐based fluorescence sensor; SEM image **(b)** and PXRD pattern **(c)** of 1‐OHP/Tb‐MOF@CP; **(d)** Fluorescence spectra of 1‐OHP/Tb‐MOF@CP in water and DPA solution (200 μM) (λ_ex_ = 278 nm, scanning range: 350–700 nm).

## Data Availability

All data will be made available upon request to the corresponding author.
