# Supplementary material for: A Ln‐MOF Sensor Based on Dual‐Signal Response and Logic Gating for the Ratio Fluorescence Detection of Anthrax Biomarkers
Source: Luminescence. 2026 May 1;41:e70493. doi: 10.1002/bio.70493 (PMC13133979; doi:10.1002/bio.70493)
Supplement: Supplementary file 1 — Table S1: Optimization of synthesis conditions for Bio‐MOF. Figure S1: (a) Particle size distribution of Bio‐MOF; (b) Fluorescence intensity of Bio‐MOF after being immersed in a DMF solution of Tb3+(0.1 mM) for different times (500 μg/mL). Figure S2: Emission spectra of 1‐OHP/Tb@Bio‐MOF composites in water and an aqueous DPA solution (1 mM) (λex = 278 nm, scanning range: 350–700 nm). Mass ratios of 1‐OHP to Tb@Bio‐MOF used for preparing the composites: (a) 1/500; (b) 1/1000; (c) 1/1333; (d) 1/2000; (e) 1/5000; (f) Chromaticity shift values. Figure S3: Excitation and emission spectra of (a) 1‐OHP (0.01 μg/mL), (b) Tb@Bio‐MOF (500 μg/mL), and (c) 1‐OHP/Tb@Bio‐MOF (500 μg/mL) (λex = 278 nm, scanning range: 200–700 nm). Table S2: Determination of DPA in human serum samples (n = 3). Table S3: Comparison of various fluorescence sensors for DPA detection. Figure S4: (a) Detection mode of the paper‐based fluorescence sensor; SEM image (b) and PXRD pattern (c) of 1‐OHP/Tb‐MOF@CP; (d) Fluorescence spectra of 1‐OHP/Tb‐MOF@CP in water and DPA solution (200 μM) (λex = 278 nm, scanning range: 350–700 nm). [file BIO-41-e70493-s001.doc]

**Supplementary Material**

## 1. Preparation of Bio-MOF

Bio-MOF was synthesized by a solvothermal method with optimized synthesis conditions^[24]^. The specific steps are as follows: First, 0.614 mmol of adenine was dissolved in 24 mL of N,N-dimethylformamide (DMF) under stirring. At the same time, in another vial, 12 mL of DMF was added, followed by the sequential addition of 0.616 mmol of zinc chloride and 0.614 mmol of sodium formate (sodium formate is used for the deprotonation of 1,3,5-benzenetricarboxylic acid (BTC)). After stirring for 10 minutes, 0.618 mmol of BTC was added. Subsequently, 9 mL of an aqueous nitric acid solution with a concentration of 66.7 mmol/L was added to the above-mentioned mixed system. After completing the above operations, the obtained mixture was transferred into a high-temperature and high-pressure reaction kettle, heated to 130 °C, and the reaction was allowed to proceed for 24 hours. Then, the reaction system was allowed to cool slowly to room temperature naturally. After the reaction was completed, the resulting white powder was collected, washed three times with DMF and ethanol respectively, and finally dried in a vacuum drying oven at 60 °C for 12 hours.

## 2. Experimental Operations for Fluorescent Sensing of DPA

When conducting the fluorescent sensing detection of DPA, take 2.5 mg of 1-OHP/Tb@Bio MOF and disperse it in 2 mL of the DPA sample solution. Subsequently, subject the mixture to ultrasonic treatment for 5 minutes to obtain a homogeneous suspension. Then, transfer this suspension into a 5-mL volumetric flask and dilute it to the mark with deionized water. At this point, the dispersion concentration of 1-OHP/Tb@Bio MOF reaches 500 μg/mL. Measure the fluorescence spectrum with an excitation wavelength of 278 nm, and record the fluorescence intensities at 388 nm (the emission wavelength corresponding to 1-OHP) and 546 nm (the emission wavelength corresponding to Tb@Bio MOF). In the selectivity and anti-interference experiments, select compounds with structures similar to DPA (such as benzoic acid, trimesic acid), amino acids present in biological samples (such as glycine, sarcosine, arginine), and anions and cations (such as K^+^, Na^+^, Ca^2+^, Mg^2+^, Cl^-^, NO_3_^2-^)^[25]^ as potential interfering substances. Add these potential interfering substances to the DPA standard solution respectively to prepare a series of sample solutions (where the concentrations of both DPA and the interfering substances are 200 μM). Thereafter, measure the fluorescence intensities of these sample solutions at 388 nm and 546 nm in the same way as described above. It should be noted that throughout the entire experimental process, the suspension needs to be shaken continuously to ensure that the MOF material is always uniformly dispersed.

## 3. Detecting DPA in serum

When detecting DPA in serum, first obtain serum samples from healthy individuals. Then, dilute the samples 200-fold with deionized water, and perform the measurement according to the method specified in item 4.2.7. Subsequently, add DPA standard solutions to the serum samples to prepare spiked blood samples at low, medium, and high concentration levels respectively. Dilute these spiked blood samples 200-fold with deionized water as well, measure them using the same method as before, and calculate the spike recovery rates.

## 4. Computational Methods

In this study, relevant calculations were carried out using the Gaussian 09 software. During the calculation process, the density functional theory (DFT) computational method was adopted. The B3LYP/6-31G^*^ basis set was selected to calculate the ground-state electronic structures of 1-OHP and DPA. Meanwhile, the time-dependent density functional theory (TDDFT) was employed, and the TDDFT/B3LYP/6-31G^*^ basis set was chosen to calculate the excited-state electronic structures of 1-OHP and DPA.

## 5.Preparation of 1-OHP/Tb@Bio MOF

**Table S1** Optimization of synthesis conditions for Bio-MOF

|  | Adenine  /mg | H_3_BTC  /mg | HCOONa  /mg | HNO_3_  /μL | Solvent/mL  （DMF/H_2_O） | T  /^o^C | Heating method | Size  /nm |
| --- | --- | --- | --- | --- | --- | --- | --- | --- |
| 1 | 83 | 130 | 41.8 | 8.87 | 24/6 | 130 | Conventional | 704 |
| 2 | 83 | 130 | 41.8 | 8.87 | 36/9 | 130 | Conventional | 474 |
| 3 | 83 | 130 | 41.8 | 8.87 | 60/15 | 130 | Conventional | 1281 |
| 4 | 83 | 130 | 41.8 | 8.87 | 120/30 | 130 | Conventional | 713 |
| 5 | 83 | 130 | 41.8 | 8.87 | 24/6 | 110 | Conventional | 779 |
| 6 | 83 | 130 | 41.8 | 8.87 | 24/6 | 90 | Conventional | 1191 |
| 7 | 83 | 130 | 41.8 | 8.87 | 24/6 | 130 | Microwave | 917 |
| 8 | 83 | 130 | 41.8 | 8.87 | 24/6 | 130 | Ultrasonic | 1307 |

Note: H_3_BTC: trimesic acid; T: heating temperature; Size: particle size of MOF; Conventional: conventional heating; Microwave: microwave heating; Ultrasonic: ultrasonic heating


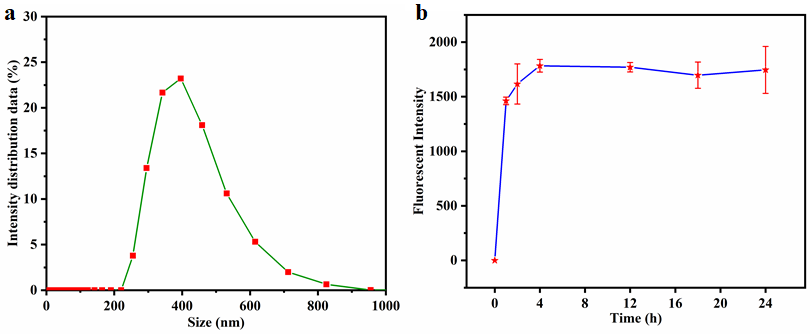


**Fig. S1** **(a)** Particle size distribution of Bio-MOF; **(b)** Fluorescence intensity of Bio-MOF after being immersed in a DMF solution of Tb^3+^(0.1 mM) for different times (500 μg/mL)


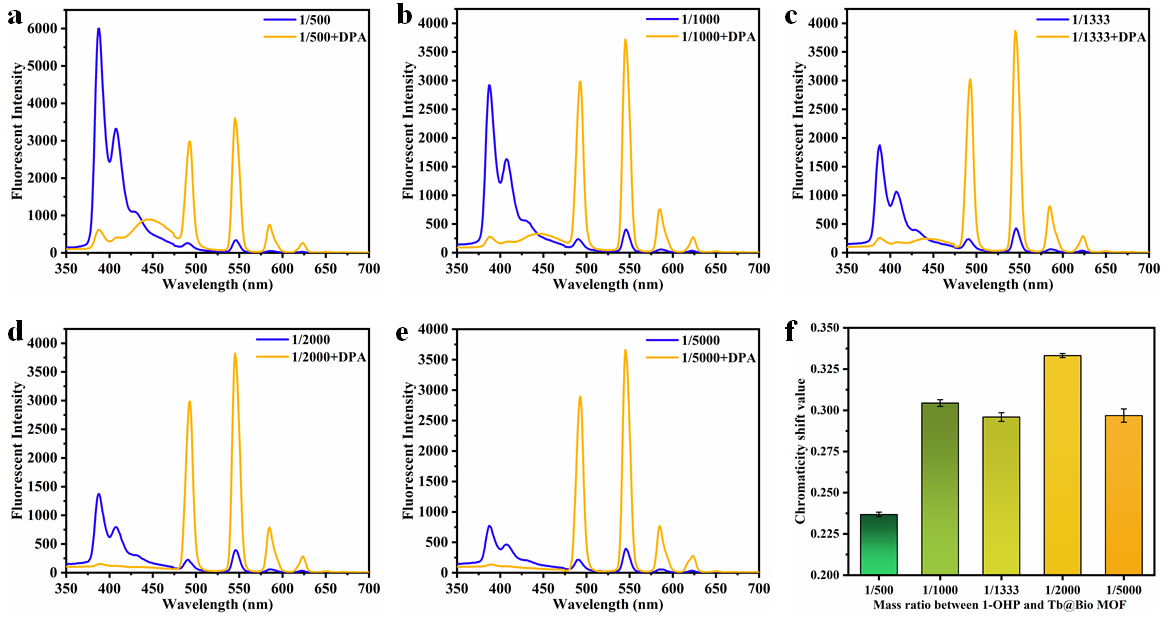


**Fig. S2** Emission spectra of 1-OHP/Tb@Bio MOF composites in water and an aqueous DPA solution (1 mM) (λ_ex_ = 278 nm, scanning range: 350–700 nm). Mass ratios of 1-OHP to Tb@Bio MOF used for preparing the composites: **(a)** 1/500; **(b)** 1/1000; **(c)** 1/1333; **(d)** 1/2000; **(e)** 1/5000; **(f)** Chromaticity shift values.

## 6. Luminescence properties of 1-OHP/Tb@Bio MOF


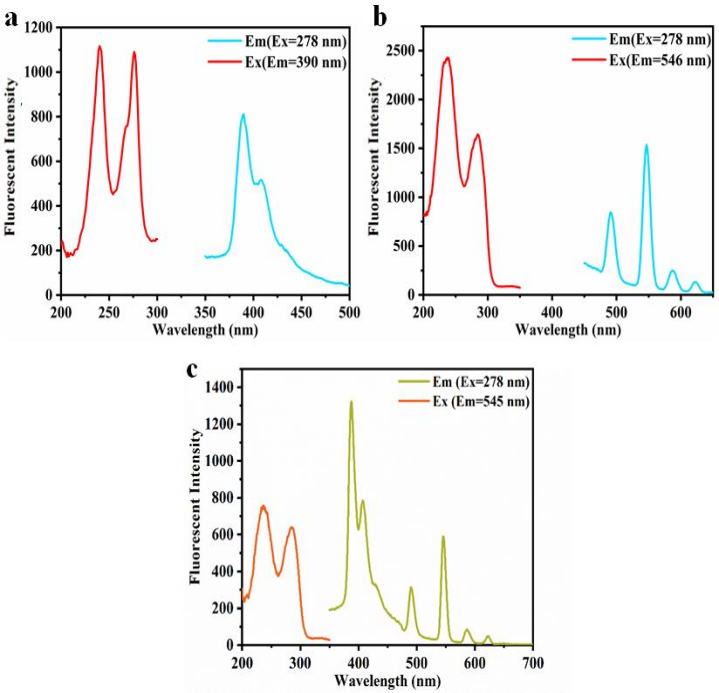


**Fig. S3** Excitation and emission spectra of **(a)** 1-OHP (0.01 μg/mL), **(b)** Tb@Bio MOF (500 μg/mL), and **(c)** 1-OHP/Tb@Bio MOF (500 μg/mL)(λ_ex_ = 278 nm, scanning range: 200–700 nm).

## 7. Determination of DPA in serum

**Table S2** Determination of DPA in human serum samples (n = 3)

| Added (μM) | Found ± SD (μM) | Recovery (%) | RSD (%) |
| --- | --- | --- | --- |
| 5 | 4.99 ± 0.30 | 99.8 | 6.01 |
| 50 | 49.03 ± 1.17 | 98.1 | 2.39 |
| 150 | 147.91 ± 4.28 | 98.0 | 2.89 |

Calibration curve equation for the DPA concentration in human serum within the range of 5-200μM: y = 0.4349 + 0.0068x (r = 0.9994).

Data are presented as mean ± standard deviation (Mean ± SD) with n=3.

According to t-test analysis, no significant difference was observed in recovery rates at each spiked level (P > 0.05);

Added: Spiked concentration of DPA in human serum samples;

Found: Concentration of DPA in spiked human serum measured by the 1-OHP/Tb@Bio MOF sensor.

## 8. Comparison with literature methods

**Table S3** Comparison of various fluorescence sensors for DPA detection

| Material | Medium | Method | Linear range | LOD | Ref. |
| --- | --- | --- | --- | --- | --- |
| Anti-DPA antibody | Phosphate buffer solution (PBS) | Enzyme-linked immunosorbent assay (ELISA) | 0.5-50 | 0.21 μM | [44] |
| Lanthanoid/Graphene | H_2_O | Single | 0-8 μM | 0.03 μM | [41] |
| MOF 1 | ethanol | Single | 0-100 μM | 3.8 μM | [2] |
| R6H@Eu(BTC) | ethanol | Ratio | 0-80 μM | 4.5 μM | [32] |
| Eu^3+^@3 | H_2_O | Ratio | 0-7 μM | 6.74 nM | [43] |
| Eu/Tb(BTC) | H_2_O | Ratio | 0-1 μM | 0.06 μnM M | [42] |
| Eu/Tb-MOFs | H_2_O | Ratio | 0-600 μM | 0.248 μM | [22] |
| Tb/Eu@bio-MOF-1 | H_2_O | Ratio | 0-1 μM | 0.34 μM | [25] |
| Ln-MOF (3) | H_2_O | Ratio | 20-100 μM | 0.172 μM | [6] |
| 1-OHP/Tb@Bio MOF | H_2_O | Ratio | 0-200 μM | 0.4 μM | this work |

Single：single emission; Ratio：ratiometric fluorescence with dual emission; MOF 1: Eu(2-amino-1,3,5-benzenetricarboxylate)(H_2_O).(H_2_O)(DMF)_2_;R6H@Eu(BTC):Rhodamine-based Eu-(1,3,5-benzenetricarboxylic acid)MOF; Eu^3+^@3: Eu^3+^@zeolite-like supramolecular assembly (2,5-thiophene-dicarboxylic acid); Eu/Tb(BTC): Eu_1_Tb_4_(isophthalic acid)_5_-MOF; Eu/Tb-MOFs: [Eu_0.1_Tb_0.9_(2,6-naphthalene-dicarboxylic acid)(H_2_O)Cl]; Tb/Eu@bio-MOF-1: Tb/Eu@bio-MOF-1 (Tb^3+^ /Eu^3+^=5.89); Ln-MOF (3): [Tb_0.43_Eu_1.57_(1,4-phenylenediacetic acid)_3_(H_2_O)](H_2_O)_2_.

## 9. Construction of paper-based DPA sensors


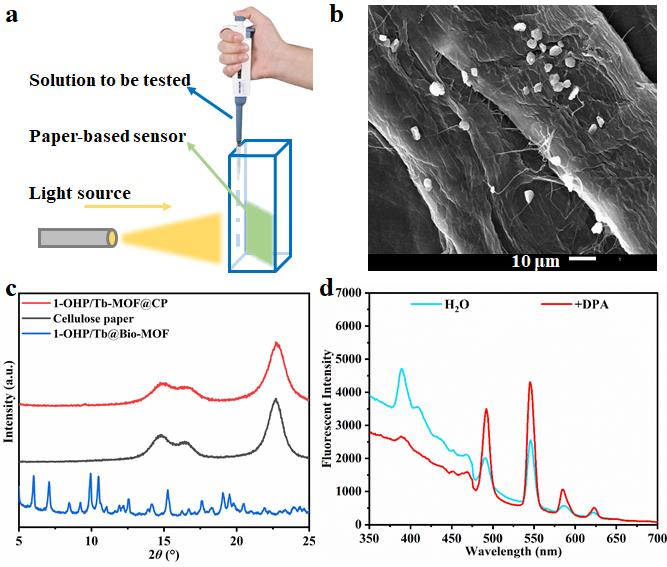


**Fig. S4 (a)** Detection mode of the paper-based fluorescence sensor; SEM image **(b)** and PXRD pattern **(c)** of 1-OHP/Tb-MOF@CP; **(d)** Fluorescence spectra of 1-OHP/Tb-MOF@CP in water and DPA solution (200 μM)(λ_ex_ = 278 nm, scanning range: 350–700 nm).
